# Expression Profiling of Differentiating Eosinophils in Bone Marrow Cultures Predicts Functional Links between MicroRNAs and Their Target mRNAs

**DOI:** 10.1371/journal.pone.0097537

**Published:** 2014-05-13

**Authors:** Ming Yang, Fiona Eyers, Yang Xiang, Man Guo, Ian G. Young, Helene F. Rosenberg, Paul S. Foster

**Affiliations:** 1 Centre for Asthma and Respiratory Disease, School of Biomedical Sciences and Pharmacy, Faculty of Health, University of Newcastle and Hunter Medical Research Institute, Callaghan, New South Wales, Australia; 2 Department of Physiology, Xiangya School of Medicine, Central South University, Changsha, Hunan, People’s Republic of China; 3 Department of Molecular Bioscience, John Curtin School of Medical Research, Australian National University, Canberra, Australian Capital Territory, Australia; 4 Inflammation Immunobiology Section, Laboratory of Allergic Diseases, National Institute of Allergy and Infectious Diseases, National Institutes of Health, Bethesda, Maryland, United States of America; Ecole Normale Superieure de Lyon, France

## Abstract

**Background:**

MicroRNAs (miRNAs) are small non-coding RNAs that regulate complex transcriptional networks underpin immune responses. However, little is known about the specific miRNA networks that control differentiation of specific leukocyte subsets. In this study, we profiled miRNA expression during differentiation of eosinophils from bone marrow (BM) progenitors (bmEos), and correlated expression with potential mRNA targets involved in crucial regulatory functions. Profiling was performed on whole BM cultures to document the dynamic changes in miRNA expression in the BM microenvironment over the differentiation period. miRNA for network analysis were identified in BM cultures enriched in differentiating eosinophils, and chosen for their potential ability to target mRNA of factors that are known to play critical roles in eosinophil differentiation pathways or cell identify.

**Methodology/Principal Findings:**

We identified 68 miRNAs with expression patterns that were up- or down- regulated 5-fold or more during bmEos differentiation. By employing TargetScan and MeSH databases, we identified 348 transcripts involved in 30 canonical pathways as potentially regulated by these miRNAs. Furthermore, by applying miRanda and Ingenuity Pathways Analysis (IPA), we identified 13 specific miRNAs that are temporally associated with the expression of IL-5Rα and CCR3 and 14 miRNAs associated with the transcription factors GATA-1/2, PU.1 and C/EBPε. We have also identified 17 miRNAs that may regulate the expression of TLRs 4 and 13 during eosinophil differentiation, although we could identify no miRNAs targeting the prominent secretory effector, eosinophil major basic protein.

**Conclusions/Significance:**

This is the first study to map changes in miRNA expression in whole BM cultures during the differentiation of eosinophils, and to predict functional links between miRNAs and their target mRNAs for the regulation of eosinophilopoiesis. Our findings provide an important resource that will promote the platform for further understanding of the role of these non-coding RNAs in the regulation of eosinophil differentiation and function.

## Introduction

Eosinophils are multifunctional granulocytes that are capable of synthesising a wide range of proinflammatory mediators [1] and are implicated in the pathogenesis of a substantial array of allergic disorders [2]. Likewise, conditions of dysregulated eosinophil growth and differentiation include complex disorders such as hypereosinophilic syndromes and eosinophilic leukaemia [3]. As such, studies that promote insight into pathways controlling eosinophil differentiation are central to understanding how these cells develop and their roles in health and disease.

Eosinophil differentiation and function is mediated by cell surface receptors that promote growth, adhesion, chemotaxis, degranulation and cell-to-cell communication [2], [4]–[6]. Although many factors contribute to the development of eosinophils, interleukin (IL) -5 plays a unique and profound role in driving differentiation and mobilisation from the bone marrow and in sustaining viability in the periphery. Likewise, eotaxins are ligands for cell surface CC-chemokine receptor 3 (CCR3), and serve to modulate eosinophil homing and accumulation in tissues both cooperatively with IL-5 and *via* IL-5-independent pathways [7], [8]. In this regard, IL-5 receptor subunit–α chain (IL-5Rα) and CCR3 play pivotal roles in orchestrating eosinophil activation, growth, differentiation and chemotaxis [2], [7], [9]–[12]. Indeed, mice deficient in IL-5, eotaxin or their respective receptors have significantly diminished numbers of eosinophils in tissues in response to inflammatory stimuli [13]–[16]. Furthermore, humanized antibodies against IL-5 (e.g. mepolizumab and reslizumab) and IL-5Rα (e.g. benralizumab) have been shown clinically to reduce circulating eosinophil numbers and/or to suppress eosinophil maturation in the bone marrow [17]–[19].

GATA family transcription factors (GATA) -1 and -2 have been implicated in driving eosinophil lineage commitment and differentiation [20]. Several lines of evidence have implicated specifically GATA-1 in guiding eosinophil development [20]–[23]. However, substantial evidence suggests that the interplay of several key transcription factors, including GATA -1 and -2, PU.1 and CCAAT/enhancer binding proteins (c/EBPs), provides unique instructive signals for the commitment and maturation of the eosinophil lineage [24]–[26]. Interestingly, c/EBPε in mouse is indispensible for terminal granulopoiesis as targeted disruption of this gene leads to failure to generate both neutrophil and eosinophil lineages [27]. However, both activator and repressor isoforms of c/EBPε in humans may specifically instruct the development of eosinophil progenitor cells into mature eosinophils [24]. Most recently, a unique role for the endoplasmic reticulum stress signaling protein, X-box protein 1 (XBP1), in promoting eosinophil lineage commitment, has been elucidated [28]. Other intracellular signaling events such as signal transduction and transcription (STAT) proteins and epigenetic regulators may also contribute to the differentiation program [29], [30].

MicroRNAs (miRNA) are approximately 22 nucleotides in length and regulate gene expression by binding to the 3′ untranslated regions of their target mRNAs to repress protein production or destabilise the target transcript [31]. The importance of miRNAs in modulating cellular function of eukaryotes (e.g. differentiation, proliferation, metabolism and apoptosis) has been established [32]–[34]. The role of miRNA in regulating the development of immune cells and controlling effective immunity is also rapidly emerging [32]. However, much less is known about the miRNA networks that control the differentiation and function of specific leukocyte subsets. Recently, miRNA have been implicated in the differentiation and control of eosinophilia in allergic inflammation, however, a detailed investigation of miRNA expression patterns employed during differentiation has not yet been performed [35]–[37]. The aim of our study is to explore miRNA expression profiling and analysis *in silico* in order to improve our understanding of the factors and circuits that are involved in the regulation eosinophil growth and differentiation. As a first approach, we have performed microarrays on whole bone marrow cultures during eosinophil differentiation to characterise the dynamics of miRNA expression in this cellular milieu. To predict functional links between miRNAs and their target mRNAs for the regulation of eosinophil differentiation, we have examined the expression profiles of miRNA in BM cultures enriched in differentiating eosinophils, which target seed sequences in critical factors known to regulate eosinophil differentiation and cell identity. Our investigation provides novel information on the miRNA expression patterns during eosinophil differentiation and as such demonstrates how specific miRNA may contribute to eosinophil identity and function.

## Materials and Methods

### Animals

Specific pathogen-free BALB/c mice (6–8 weeks) were obtained from the animal services unit of the University of Newcastle. All experiments were performed with approval from the Animal Ethics Committee of the University of Newcastle (Permit Number: A-2010-136). All surgery was performed under sodium pentobarbital anesthesia, and all efforts were made to minimize suffering.

### Culture and Identification of Bone Marrow-derived Eosinophils

Bone marrow-derived eosinophils (bmEos) were differentiated and examined as previously described [21]. Briefly, mouse femurs were flushed with 5 ml ice-cold Hank’s buffered saline (HBSS) through a 70 µm cell strainer. After lysis of red blood cells and washing with phosphate buffered saline (PBS) bone marrow cells were cultured at a concentration of 1×10^6^ cells/ml in RPMI-1640 medium (Invitrogen) containing 20% fetal calf serum (FCS), 4 mM glutamine, 100 U/ml penicillin, 100 µg/ml streptomycin, 25 mM hydroxyethyl piperazineethanesulfonic acid, 50 µM 2-mercaptoethanol, 1x non-essential amino acids and 1 mM sodium pyruvate. Culture medium was supplemented with 100 ng/ml stem-cell factor (SCF; PeproTech) and 100 ng/ml FLT3-Ligand (Flt3L; PeproTech) at 37°C in a humidified atmosphere of 5% CO_2_ and 95% air. On day 4, the media containing SCF and Flt3L was replaced with media supplemented with 10 ng/ml recombinant mouse IL-5 (R&D Systems). Then, every second day from day 4, one half of the media was replaced with fresh media containing IL-5 and the cell concentration was maintained at 1×10^6^ cells/ml. Cells were collected every second day for miRNA microarray and qPCR. The purity of bmEos was determined using flow cytometry and Giemsa staining.

### Giemsa Staining

5×10^5^ bmEos were centrifuged in 100 µl aliquots onto clean glass slides for 5 min at 300× g using a Cytospin centrifuge. Cytospin preparations were air dried and fixed followed by staining with Giemsa. Eosinophils were determined morphologically as previously described [21], [35].

### Flow Cytometry

Cells (3×10^5^) were incubated first with mouse FcBlock (2.4G2; BD PharMingen, San Diego, CA, USA) to inhibit nonspecific binding of antibodies. After washing, cells were stained with anti-Siglec F, anti-Gr-1, anti-CD11b, anti-CD11c and respective isotype controls (BD Biosciences, PharMingen). Numbers of positive cells were quantified by flow cytometry (FACSCanto flow cytometer, BD Biosciences, San Jose, CA). Eosinophils were identified as SiglecF^+^Gr-1^+^CD11b^+^CD11c^-^ [21], [38]. Data were collected on a FACSCanto flow cytometer and analysed with FlowJo software (version 10, Tree Star, Inc).

### miRNA Microarray

Total RNA was extracted from bone marrow cells and bmEos using TRIzol reagents (Life Technologies) and miRNA microarray was performed as previously reported [35]. Briefly, the Agilent spike-In control was added to 100 ng RNA, which was dephosphorylated by incubating the samples at 37°C for 30 minutes followed by ligation of Cy3 using the Complete Labelling and Hybridisation c-Kit (Agilent). Following ligation and drying, the Cy3-labelled RNA samples were hybridized for 20 h at 55°C to Agilent 8×15K mouse microRNA array slides (AMADID 21828), which included 627 mouse miRNA and 39 mouse viral miRNA from the Sanger database 12.0. After washing with Agilent gene expression wash buffers, the hybridized microarrays were scanned on a High Resolution C scanner (Agilent). Data were extracted from scanned microarrays using Feature Extraction software (version 10.7.3.1). The fluorescence index of each miRNA at different timepoints was further normalized to that of the respective miRNAs in control group (isolated bone marrow cells). The normalized microarray data were managed and analyzed by GeneSpring (Agilent). MicroArray data have been deposited into ArrayExpress (http://www.ebi.ac.uk/arrayexpress/). The accession number is E-MTAB-2442.

### miRNA Quantitative Polymerase Chain Reaction

miRNA quantitative polymerase chain reactions (qPCR) were performed using the TaqMan MicroRNA reverse transcription kit (Life Technologies), Taqman MicroRNA qPCR assays (Life Technologies) and TaqMan Universal PCR Master Mix, as previously described [35]. qPCR reactions were performed according to the manufacturers’ suggested conditions. Sno202 was used as a housekeeping control RNA in the experiments. Relative expression was calculated using the 2^−ΔΔCt^ method.

### miRNA Target Analysis

For prediction of target genes of differentially expressed miRNAs, we first used TargetScan 6.1 (http://www.targetscan.org/) to identify the potential mRNA targets. MeSH database (http://www.nlm.nih.gov/mesh/meshhome.html) was then employed to identify the molecules relevant to eosinophil biology by exact syntax matching. MiRanda (http://www.microrna.org/) was also used to refine the predicted targets. Ingenuity Pathways Analysis (Ingenuity Systems, Redwood City, CA) software was further used to identify canonical signaling pathways containing the miRNA-associated eosinophil-associated molecules, and to establish network connections between miRNAs and their respective predicted targets.

### mRNA Quantitative PCR

The method for qPCR has been described in detail elsewhere [9], [23]. Briefly, total RNA was isolated from bone marrow cells and bmEos from week 2 to week 6 with TriReagent (Life Technologies), and reverse-transcribed using M-MLV Reverse Transcriptase (Life Technologies). qPCR was performed using an ABI Viia7 qpcr machine (Life Technologies). Amplicons were detected using SYBR green and expression was normalized to hypoxanthine-guanine phosphoribosyl transferase (HPRT). Primers sequences are shown in Table S1.

### Statistical Analysis

An initial one-way ANOVA was followed by appropriate comparisons to test for differences between means of groups. Values are reported as the means ± SEM for each experimental group. The number of samples at each time-point ranged from 4 to 6. Differences in means were considered significant if p was <0.05. For regression analyses, significance was determined by using Pearson correlation tests. Pearson statistics are reported in Table S8 in File S1.

## Results

### Generation of bmEos

**Figure 1:**
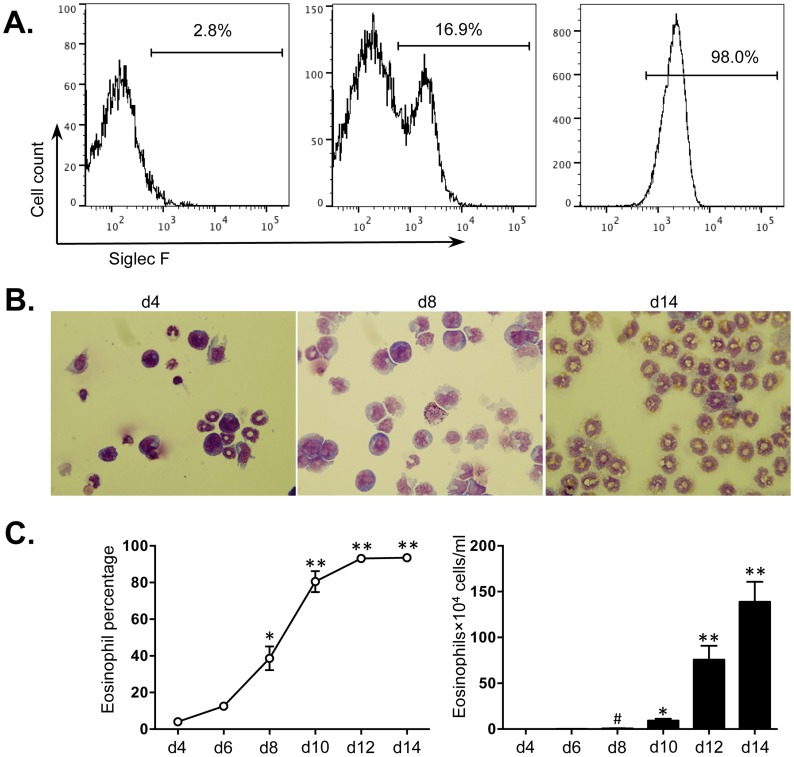
Expansion of bmEos *ex vivo*. Bone marrow cells from BALB/c mice were cultured for 14 days, see Methods and samples taken from day 4 to day 14 in cultures grown in the presence of IL-5. Eosinophils were identified by (A) flow cytometry (SiglecF^+^Gr-1^−^CD11b^+^CD11c^−^), (B) light microscopy with Giemsa staining (100×) and (C) the percentages and numbers of bmEos were determined using a haemocytometer. Values are presented as mean ±SEM (n = 4∼6), **P<0.01 (d10, d12 or d14 vs. other groups). *P<0.05 (vs. d4, d6 or d8). # P<0.05 (vs. d4 or d6).

Isolated bone marrow cells from mice were cultured for 14 days as described in Methods. On day 4, before the addition of IL-5, and on day 8 and 14 in the presence of IL-5, the percentage of bmEos was first determined by FACS; the characteristic morphological features of bmEos, including bilobed nucleus and red-staining cytoplasmic granules, were identified by Giemsa staining. The percentage of bmEos (SiglecF^+^Gr-1^−^CD11b^+^CD11c^−^) increased from 2.8% on day 4 to 98.0% by day 14 (Fig. 1A). These percentages were also observed with Giemsa staining of cytospin preparations (Fig. 1B and C). The eosinophil population underwent significant expansion after day 8 in culture. The numbers of bmEos were increased approximately 320-fold from 0.5±0.4×10^4^ cells/ml at day 4 to 139.0±22.0×10^4^ cells/ml on day 14 (Fig. 1C).

### Distinct miRNA Expression Profile during Eosinophil Development

**Figure 2:**
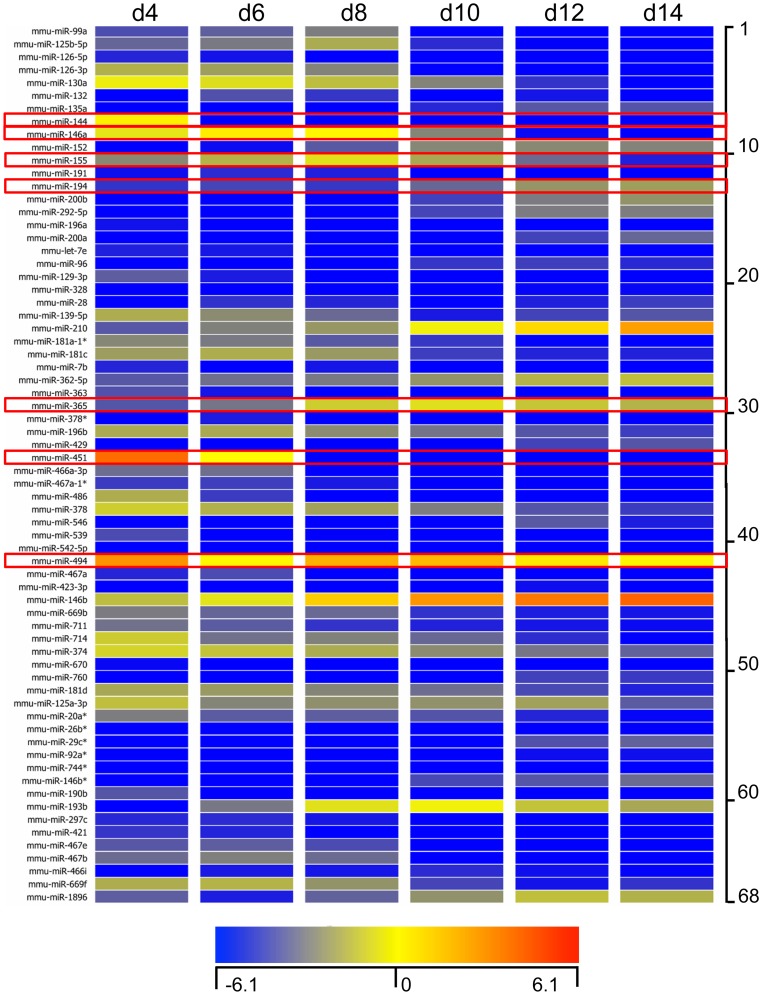
Expression levels of miRNA during bmEos differentiation. Bone marrow cells from BALB/c mice were cultured for 14 days. Heat map representation of expression levels of miRNA that were up-regulated or down-regulated by more than 5-fold from day 4 to day14 in the presence of IL-5. The fluorescence index of each miRNA at different timepoints was further normalized to that of the respective miRNAs in the control group (isolated bone marrow cells). The normalized microarray data were managed and analyzed by GeneSpring (Agilent). Data represent three independent bmEos cultures. Scale ranges from a signal value of −6.1(blue) to 6.1(red). Some of miRNAs were selected to further confirm the efficacy of miRNA array.

**Figure 3:**
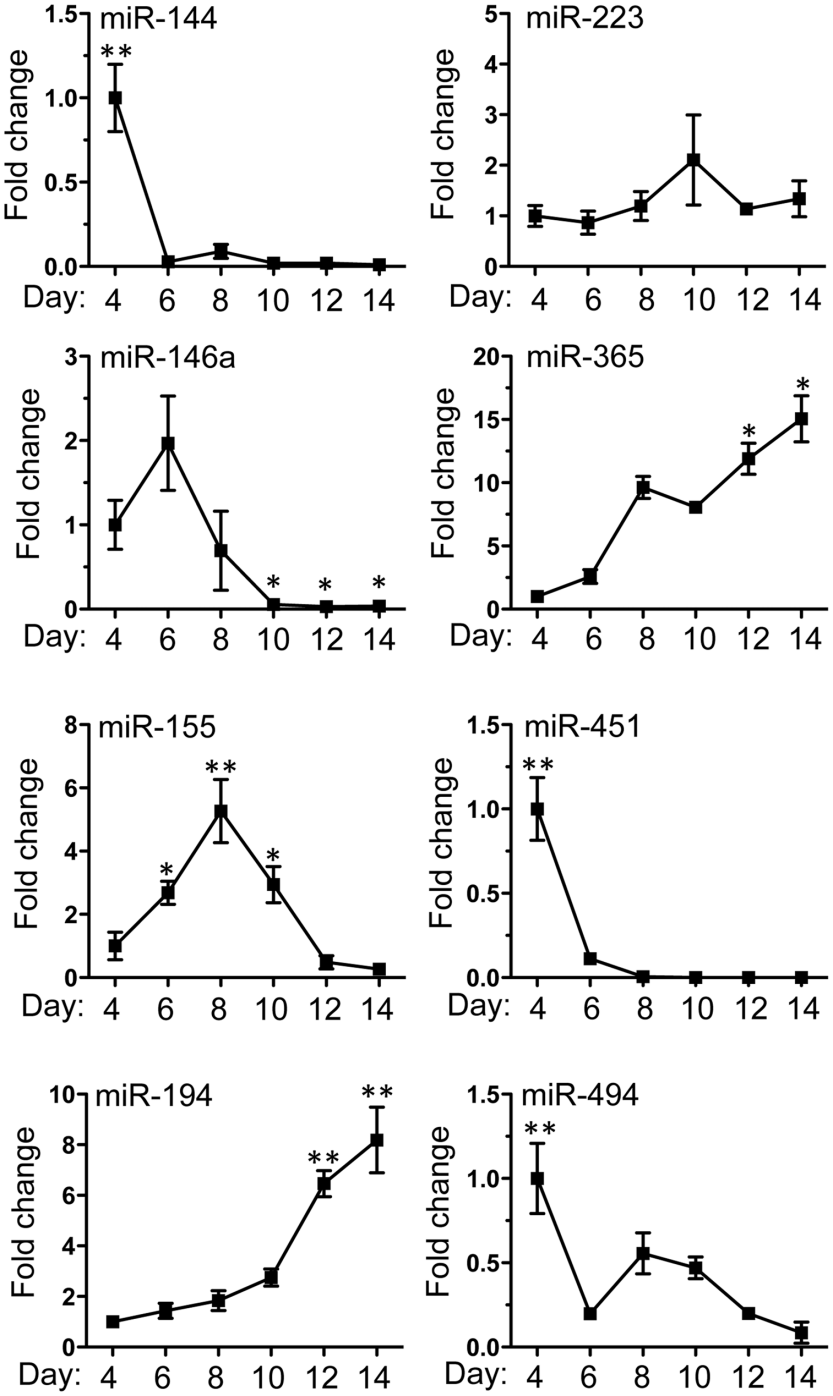
Confirmation of miRNA array expression by Taqman quantitative PCR. 8 miRNAs (miRNA -144, -223, -146a, -365, -155, -451, -194 and -494) were selected to verify the expression profile of the miRNA array. Samples were RNA isolated from cultured bone marrow cells from day 4 to day14 in the presence of IL-5, and represent three independent cultures. Values are presented as mean ±SEM (n = 4∼6), **P<0.05 (vs. other groups). *P<0.05 (vs. d4).

Given the broad involvement of miRNAs in the regulation of post-transcriptional gene expression, we next determined the miRNA profile of bmEos from day 4 to day 14. Total RNA was isolated from cells sampled every second day and screened as described in Methods. A total of 68 unique miRNAs were selected based on 5-fold increased or decreased expression during bmEos differentiation. Among them, 42 exhibited decreased and 26 exhibited increased expression, respectively (Fig. 2). Detailed information describing these miRNAs is included in Table S2 in File S1. We confirmed the differential expression of 7 of the 68 miRNAs with qPCR (Fig. 3). These specific miRNAs have been linked to leukocyte development and inflammation [39]–[45]. We also examined the expression levels of miR-223, which has been reported to be a critical regulator of myeloid development [46].; interestingly, levels of this miRNA remained largely unchanged over the differentiation period.

### Critical Transcriptional Regulators for Eosinophilopoiesis are Targeted by Distinct Sets of miRNAs

**Figure 4:**
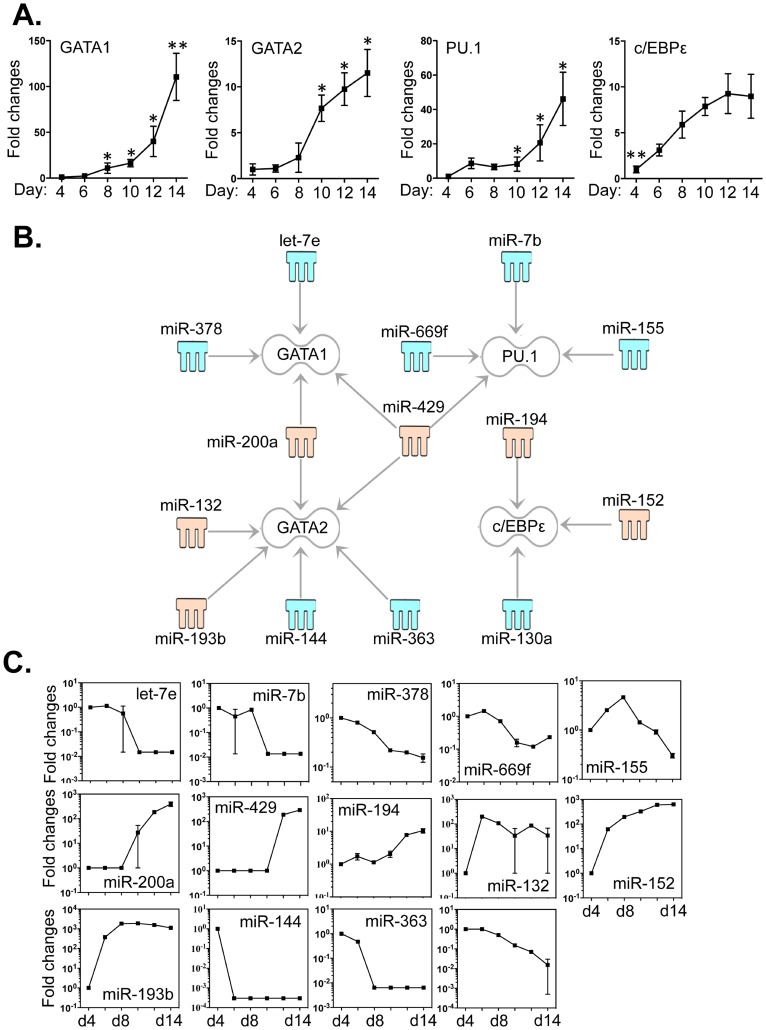
Expression of GATA -1/2, PU.1 and c/EBPε correlated with the expression levels of miRNAs that potentially target these transcripts. Bone marrow cells were cultured as described in Methods and RNA samples were extracted from cells taken from day 4 to day14 which had been grown in the presence of IL-5. *A*. Expression levels of GATA -1/2, PU.1 and c/EBPε were determined by qPCR. *B*. Potential miRNAs targeting the 3′-UTR of the four transcription factors were identified by TargetScan, the Miranda database and IPA ingenuity system. Blue represents decreased expression of miRNAs, whereas yellow is for increased expressed miRNAs. *C*. The fold changes of potential regulating miRNAs were calculated based on the fluorescence index of each miRNA at different time-points, after normalization to that of the respective miRNAs in the control group (isolated bone marrow cells). Data represent three independent eosinophil cell cultures. Values are presented as mean ±SEM (n = 4∼6), **P<0.05 (vs. other groups). *P<0.05 (vs. d4).

The differentiation of eosinophils is critically determined by a coordinated interaction between GATA -1/2, PU.1 and c/EBPε [25]. Thus, we evaluated the expression levels of these factors by qPCR and correlated these levels to those of the 68 differentially expressed miRNAs (Fig. 2). As anticipated, the expression levels of the four transcription factors were significantly enhanced (Fig. 4A), as previously reported, during differentiation [22], [23], [25], [47]. With IPA software and the miRanda database, we identified 14 miRNAs that may have an impact on the expression of these four crucial transcriptional regulators (Fig. 4B). We correlated the expression levels of these miRNAs to GATA1 (let-7e, miR -200a, -378 and -429), GATA2 (miR -132, -144, -193b, -200a, -363 and -429), PU.1 (miR -7b, -155, -429 and -669f) and c/EBPε (miR -130a, -152 and -194) (Fig. 4C). Both miR-200a and miR-429 are linked to GATA -1 and -2; miR-429 is particularly notable, as it also linked to expression of a third transcription factor, PU.1. The 3′-UTR binding sites of miRNAs that target these mRNAs are shown in Table S3 in File S2.

### Analysis of Eosinophil-related Molecules and Canonical Pathways Potentially Regulated by the miRNAs

**Figure 5:**
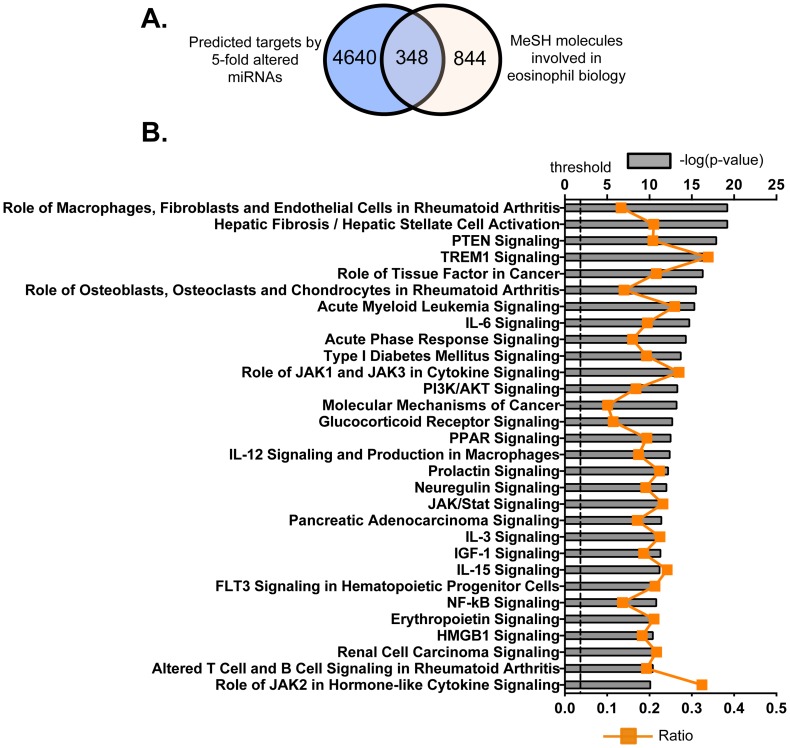
Potential molecules and top canonical pathways that were predicted and targeted by the miRNAs that exhibited 5-fold changes in expression. *A*. Target prediction by TargetScan database (http://www.targetscan.org/) was established on sequence data complementarity to target 3′UTR sites. Target molecules, associated with eosinophil biology, were identified by exact syntax matching in the MeSH database. (http://www.nlm.nih.gov/MeSH/MeSHhome.html). *B*. Top 30 canonical pathways that consist of the putatively selected 348 molecules as identified by IPA. The significance of association between selected genes and canonical pathway was evaluated by a right-tailed Fisher’s exact test to calculate a p value determining the probability that the association is not explained by chance alone (grey bars, upper y-axis). Ratios referring to the proportion of selected genes from a pathway related to the total number of molecules that make up that particular pathway were also displayed (line graph, bottom y-axis).

By using TargetScan (version 6.1), we identified consensus mRNA targets of these 68 miRNAs. TargetScan is a search engine for predicted targets of miRNA in eukaryotes. It searches for the conserved and/or non-conserved 8- and 7-mer sites, which share sequence homology with each miRNA, that are located in the 3′-UTR regions of mRNAs [48]. Predictions are then ranked by their probability of targeting the mRNA transcript [49]. In this study, the 95^th^ percentile was used to predict the potential targets by these 68 miRNAs with TargetScan. We identified 4988 mRNA transcripts that had the potential to be targeted by this group of miRNAs (Fig. 5A). The target mRNAs were further refined by searching the MeSH database, in an effort to link gene expression data with known eosinophil-related pathways, diseases and phenotypes. There were 1192 mRNAs identified by MeSH database as containing the exact terms “eosinophils, eosinophilia, IL-3, IL-5, eosinophil peroxidase, eosinophil cationic protein, eosinophil granule proteins, eosinophil major basic protein or eosinophil-derived neurotoxin” with a link to at least one PubMed-affiliated reference. We then plotted differences in distribution of mRNAs predicted by TargetScan and MeSH databases, and found that, of the original 4988 transcripts, we could identify 348 that were associated with eosinophil biology and targeted by members of the 68 miRNAs that were identified by both search methods (Fig. 5A and Table S4 in File S1).

To gain further understanding of the way in which the 348 eosinophil-associated mRNAs contribute to intracellular events in eosinophils, these genes were then classified according to signaling pathways using the IPA Ingenuity system; the top 30 canonical pathways were listed (Fig. 5B; Table S5 in File S1). There are 140 molecules (40.2% of the 348 eosinophil-associated transcripts) that are included within these top 30 canonical pathways. Although many of the pathways are commonly involved in cell death and survival or proinflammatory activity, these pathways may also contribute to eosinophil differentiation and function. For example, Glucocorticoid Receptor Signaling may contribute to eosinophil viability [50] and PI3/AKT Signaling is critical for degranulation [51]. Although evidence is limited, pathways such as IL-3, Insulin-like growth factor 1 (IGF-1), IL-15, FLT3 Signaling in Hematopoietic Progenitor Cells, Erythropoietin and Role of JAK2 in Hormone-like Cytokine Signaling pathways may serve to regulate eosinophil differentiation and proliferation. The well-known inflammatory pathways such as IL-6, Acute Phase Response, Peroxisome Proliferator-Activated Receptor (PPAR), IL-12 and Nuclear Factor- κB (NF- κB) likewise regulate the production of proinflammatory factors by eosinophils. These cells may also use Triggering Receptor Expressed on Myeloid Cells 1 (TREM1) pathway to respond to infection, as TREM1 pathway is critical for the regulation of acute inflammatory responses to microbial products [52]. Interestingly, eosinophils may contribute to the development of rheumatoid arthritis [53], [54], as the three signaling pathways, Role of Macrophages/Fibroblast and Endothelial Cell, Altered T Cell and B Cell in Rheumatoid Arthritis and Role of Macrophages/Fibroblast and Endothelial Cell in Rheumatoid Arthritis, have been linked to pathogenesis of this disease. Collectively, these data suggest important roles for miRNAs in the regulation of both the biological function and cell death/survival of bmEos.

### Multiple miRNAs are Linked to the Expression of IL-5Rα Chain and CCR3

**Figure 6:**
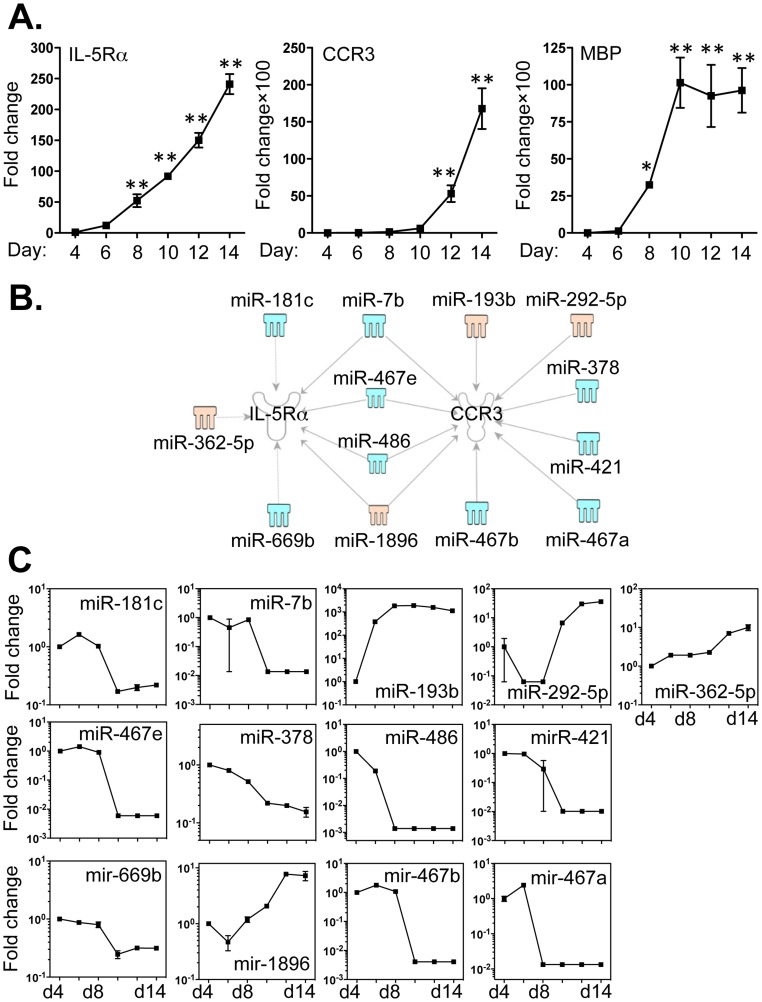
Expression levels of IL-5Rα, CCR3 and MBP correlated with the expression of miRNAs that potentially target these transcripts. Bone marrow cells were cultured as described in Methods and RNA samples were extracted from day 4 to day14 from cells grown in the presence of IL-5. *A*. Expression levels of IL-5Rα, CCR3 and MBP were determined by qPCR. *B*. Potential miRNAs targeting the 3′-UTR of IL-5Rα and CCR3 were identified by TargetScan and MiRanda database and IPA ingenuity system. Blue represents decreased expression of miRNAs, whereas yellow is for miRNAs with increased expression. *C*. The fold changes of potential regulating miRNAs were calculated based on the fluorescence index of each miRNA at different time-points, after normalization to that of the respective miRNAs in the control group (isolated bone marrow cells). Data represent three independent eosinophil cell cultures. Values are presented as mean ±SEM (n = 4∼6), **P<0.001 (vs. other groups). *P<0.01 (vs. BM).

As IL-5Rα, CCR3 and the secretory mediator, major basic protein (MBP) are critical signature molecules for eosinophils, we examined the expression of these factors in bmEos cultures from day 4 to day 14 by qPCR (Fig. 6A). Relative expression of transcripts encoding all three factors underwent significant increases between day 4 and day 14, corresponding to the profound increase in eosinophil number during this time period. We used IPA software and the miRanda database to examine the relationships between miRNAs and these specific transcripts, in order to determine whether any of the 68 differentially expressed miRNAs (Fig. 2) might have binding sequences that could target their respective 3′-UTRs. Interestingly, none of the aforementioned 68 miRNAs has any potential to regulate MBP or eosinophil-associated ribonuclease (EAR) 1 and 2 (data not shown). By contrast, IL-5Rα chain is linked to 7 miRNAs; expression of 5 of these miRNAs undergoes a decrease (miR -7b, -181c, -467e, -486 and -669b) and 2 (miR -362-5p and -1896) were increased (Fig. 5B and C). CCR3 may be targeted by 10 miRNAs, of which 7 (miR -7b, -378, -421, -467a, -467b, -467e and -486) were decreased and 3 (miR -193b, -292-5p and -1896) were increased (Fig. 6B and C). Moreover, four miRNAs (miR -7b, -467e, -486 and -1896) may regulate the expression of both the IL-5Rα chain and CCR3. The 3′-UTR binding sites of these miRNAs are included in Table S6 in File S2.

### Association between miRNAs and Expression of Genes Encoding Toll-like Receptors (TLRs) by bmEos

**Figure 7:**
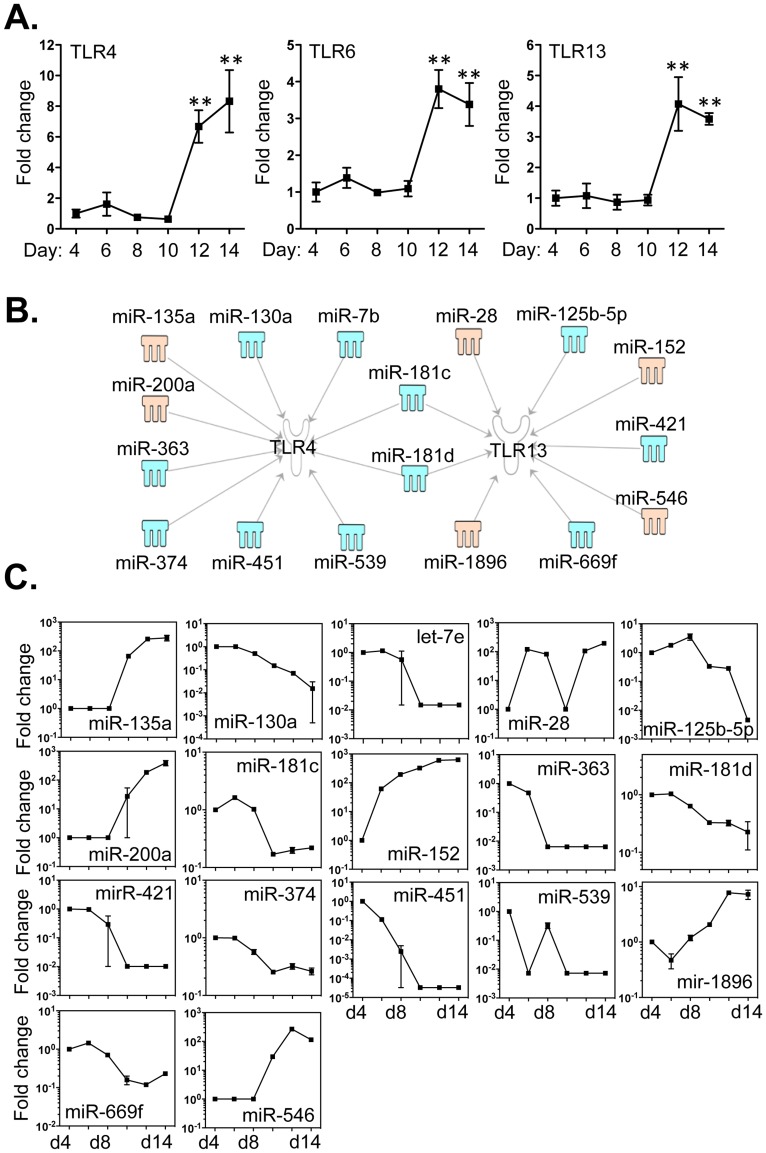
Expression of TLR4, TLR6 and TLR13 correlated with the expression of miRNAs that potentially target these transcripts. Bone marrow cells were cultured as described in Methods and RNA samples were extracted from day 4 to day14 from cells grown in the presence of IL-5. *A*. Expression levels of TLR4, TLR6 and TLR13 were determined by qPCR. *B*. Potential miRNAs targeting the 3′-UTR of TLR4, TLR6 and TLR13 were identified by TargetScan, the MiRanda database and IPA ingenuity system. Blue represents decreased expression of miRNAs, whereas yellow is for miRNAs with increased expression. *C*. The fold changes of potential regulating miRNAs were calculated based on the fluorescence index of each miRNA at different time-points, after normalization to that of the respective miRNAs in the control group (isolated bone marrow cells). Data represent three independent eosinophil cell cultures. Values are presented as mean ±SEM (n = 4∼6), **P<0.001 (vs. d4, d6, d8 or d10).

TLRs are vital innate immune receptors that detect and respond to signals from infectious pathogens. Studies carried out *in vitro* indicate that eosinophils express numerous TLRs [55]–[57]. We detected increased expression of transcripts encoding TLRs -4, -6 and -13 between days 4 and 14, while transcripts encoding TLR -1, -2, -3, -5, -7, -8, -9, -11 and -12 were decreased (Fig. 7A and Fig. S1). By employing IPA software and miRanda databases, 10 miRNAs were directly associated with mRNA encoding TLR4, of which 8 (miR -7b, -130a, -181c, -181d, -363, -374, -451 and -539) were decreased and 2 (miR -135a and -200a) were increased (Fig. 7 B and C). TLR13 was associated with the decreased expression of 5 miRNAs (miR -125b-5p, -181c, -181d, -421 and -669f) and the increased expression of 4 miRNAs (miR -28, -152, -546 and -1896; Fig. 7B and C). Moreover, miR-181c and miR-181d have the potential to regulate the expression of both TLR4 and TLR13. By contrast, none of the aforementioned 68 miRNAs has the potential to regulate the other TLRs. The 3′-UTR binding sites of miRNAs that target these mRNAs are shown in Table S7 in File S2.

## Discussion

Our study was undertaken in order to examine miRNA expression in bmEos during differentiation from a global perspective starting within the cellular milieu of the BM, and then to correlate miRNAs with expression of key eosinophil signature transcripts in cultures enriched in differentiating eosinophils. We have established *ex vivo* culture of bmEos and determined the expression of characteristic receptors and transcriptional regulators during their development. The miRNA expression profile was determined and correlated to expression of molecules known to be critical to eosinophil development by stringent statistical and pathways analysis. With these approaches, we report a unique miRNA network that is closely linked to eosinophil differentiation programe.

Although mouse models are widely used to elucidate the pathogenesis of human disease, their effectiveness towards interpreting clinical observations remains a subject of debate [58], [59]. In particular, the differences between human and mouse eosinophils are often used to explain the failure of mouse models to reproduce important features of human allergic and parasitic diseases. While it is true that significant differences between human and mouse eosinophils exist [60], this does not necessarily imply global and fundamental disparities in differentiation and overall function [60]. Indeed, both species share similar signaling mechanisms leading to the generation of terminally differentiated eosinophils from committed progenitors [60]. For example, IL-3, IL-5 and GM-CSF are eosinophilopoietic cytokines that critically regulate the survival, expansion and differentiation of eosinophils in both species [2], [6], [50]. The progenitors from both human and mouse show a similar expression pattern of GATA family transcription factors as well as other factors known to play key roles in eosinophil differentiation, including PU.1 and c/EBP proteins [24]–[26].

Eosinophil lineage progenitors are derived from hematopoietic stem cells. Although it is recognized that a unique combinatorial interaction of GATA -1 and -2, PU.1 and c/EBPε may provide essential signals for the maturation [24]–[26], other factors may also contribute to the differentiation. In this context, miRNAs provide an additional but critical level of control by modulating mRNA translation. Because miRNAs are not perfectly complementary to their targets, each is capable of regulating a large number of mRNAs. As such, miRNAs can potentially modulate entire transcriptional programs, although key individual targets may also be critically important [32]–[34]. Our results suggest that the four key transcriptional factors are differentially targeted by distinct groups of miRNAs (Fig. 4). To further validate the relationships between miRNA and target mRNA, we cloned the 3′UTR regions of GATA-1 into dual-fluorescence luciferase reporter constructs (see Method S1). Co-transfection of luciferase constructs with the mimics of the four miRNAs resulted in a dose-dependent decreases in luciferase activity, compared to control mimic, demonstrating that the four miRNAs directly target GATA-1 transcript (Fig. S2). Notably, let-7e, miR-7b, -144 and -130a, that were each ∼100-fold decreased during eosinophil hematopoiesis, are associated with the increased levels of transcript encoding GATA-1, PU.1, GATA-1 and c/EBPε, respectively. Notable, change of miR-130a was strongly correlated to that of c/EBPε by Pearson correlation test (P<0.0005, r = −0.97, Table S8 in File S1). In addition, miR -200a and -429 potentially regulate multiple distinct transcription factors (Fig. 4B). Interestingly, these two miRNAs belong to the same miR-200 family that play an essential role in the suppression of tumors by inhibiting epithelial mesenchymal transition [61], suggesting their roles (together with other miRNAs) in controlling potential over-expression of the targets and maintaining a fine balance between the four key transcription factors.

Moreover, Lu *et al* (with C57BL/6 mice) have identified that miR-21 regulates the development of eosinophils by modulating the growth of eosinophil progenitors [62]. However, we could not identify the significant changes of miR-21 in our list of miRNAs. Considering that we used BALB/c background mice, we speculate that the difference of the two studies is likely attributed to the strain difference. Although miRNAs are highly conserved in animals, the profiles of miRNA expression were previously found to be significantly different across inbred mouse strains [63]. Furthermore, hematopoietic stem- and progenitor- cells exhibit striking differences in genetic traits between mouse strains [64], [65]. Recently, a study of mouse progenitor cells also indicates that miRNA profiles are strain-dependent [66]. Nevertheless, Gerrits *et al* have found an evolutionary conserved miRNA cluster containing miR -99b, let-7e and miR-125a [66]. Notably, these three miRNAs are highly expressed in the progenitor cells of different mice strains but down-regulated during differentiation. Similarly, we have also found that the expression of let-7e and miR-125a were significantly decreased during eosinophil maturation. By contrast to miR-99b, we have shown that miR-99a expression was significantly reduced upon eosinophil differentiation. Interestingly, both miR-99a and miR-99b belong to the same miR-99 family which regulates cell proliferation by targeting Akt/mTOR (mammalian target of rapamycin) signalling [67]. These studies strongly support our observations and that of others in terms of function and strain dependence.

Most recently, Bettigole *et al* showed that X-box binding protein 1 (XBP1) selectively determines eosinophil development, independently from other known eosinophil-related transcription factors including GATA-1 [28]. Interestingly, we have also demonstrated that three miRNAs -(e.g. miR -125a-3p, -196a and -196b)- with decreased profiles may also target XBP1 transcripts, although we didn’t detect altered expression of XBP1 during bmEos differentiation (data not shown). These results suggest that the aforementioned miRNAs may control the translation of their target instead of degrading XBP1 transcript and, as such, they represent a distinct network in control of eosinophil maturation.

To investigate further the connectivity of miRNAs and other intracellular signaling pathways, we generated a list of 348 potential mRNAs that associated with eosinophil biology and are targeted by the miRNAs, by cross-comparison with TargetScan and MeSH database. With IPA Ingenuity system, we further showed that 40.2% of miRNAs-targeted mRNAs were included within the top 30 canonical signaling pathways, among which were molecules controlling cell cycle, growth and death, and cell activation (Fig. 5). For example, PTEN signaling and PI3K/AKT signaling are closely paired, and serve to balance one another in order to regulate cell proliferation. Impairment of PTEN signaling may lead to PI3K/AKT hyperreactivity, resulting reduced apoptosis and unchecked cell proliferation [68]. Furthermore, miRNA may also target other pathways such as IL-3, IGF-1, IL-15 and FLT3 signaling that may contribute to the early eosinophil progenitor commitment. Interestingly, miRNAs are associated with HMGB1 pathway, indicating that miRNAs indirectly regulate chromatin remodelling. In addition, inflammatory pathways such as IL-6, IL-12, JAK/STAT and NF-kB signalings may be closely modulated by miRNAs.

Interestingly, among the 348 potential miRNA targets, IL-5Rα and CCR3 are well-known signature receptors of eosinophils [2], [4], [5]. The expression of IL-5Rα and CCR3 were increased almost 250-fold and 180-fold respectively, from day 4 to day 14 of culture (Fig. 6A). These results are consistent with the expansion of the SiglecF^+^Gr-1^+^CD11b^+^CD11c^−^ bmEos population. Although MBP transcripts were also increased about 100-fold, none of 68 miRNAs identified here are known to target this molecule. Furthermore, none of these miRNAs are associated with EAR 1 and 2 transcripts encoding the eosinophil associated ribonucleases, which are the mouse orthologs of eosinophil cationic protein (ECP) and eosinophil-derived neurotoxin (EDN). Interestingly, four miRNAs (miR -7b, -467e, -486 and -1896) were closely associated with the expression of both IL-5Rα and CCR3. Moreover, several miRNAs (e.g. miR -193b, -292-5p, -362-5p, -467a -467b, -467e and -486 -1896) recognize the multiple sites in the IL-5Rα and/or CCR3 3′-UTR (Table S6 in File S2). Among these miRNAs, the expressions of four miRNAs (miR-7b (P<0.05, r = −0.7617); miR-181c (P<0.05, r = −0.7719); miR-467e (P<0.05, r = −0.826) and miR-669b (P<0.05, r = −0.8171)) were negatively correlated to the expression of IL-5Rα by Pearson correlation test (Table S8 in File S1).

Eosinophils interact with the environment *via* releasing their granule contents including MBP, ECP and EDN [2]. Recent findings suggest that eosinophils interact with bacteria by producing neutrophil-extracellar traps (NETs) structures composed of mitochondrial DNA, MBP and ECP [69]. Indeed, stimulation of eosinophils with lipopolysaccharide (LPS) promotes the cells to produce tumor necrosis factor α and eosinophil cationic protein in a dose-dependent manner. Likewise, studies carried out *in vivo* suggest that activation of TLR7/MyD88 is essential for eosinophil-mediated clearance of virion challenge [57]. However, how eosinophils specifically recognize infectious targets remains uncertain. In this context, TLRs are among the most important pattern recognition receptors and they may act as infection sensors for eosinophils [55]–[57]. Indeed, there are reports showing that human eosinophils constitutively express TLR transcripts (e.g. TLR -1, -4, -7, -9 and -10), and mouse eosinophils can express both surface and intracellular TLR receptors (TLR -3, -4 and -7) [56], [57]. To determine if any of the 68 miRNAs are linked to TLRs, we first determined the expression levels of all twelve mouse TLRs and found that three TLRs (TLR4, TLR6 and TLR13) are increased while those encoding the remaining TLRs decrease during bmEos differentiation (Fig. 7). TLR13 is newly identified as a molecular sensor for bacterial 23S rRNA [70]. TLR13 is an intracellular receptor, belonging to TLR11 family [71]. Currently, it is believed that intracellular TLRs recognize nucleic acid sensors [72], suggesting a likely role of TLR13 in anti-viral infections. There is no identified human ortholog of this receptor. It is interesting to note that the TLR13 transcript is identified in eosinophils; whether these cells employ TLR13 to regulate anti-bacterial infection requires further studies. In order to explore the connections between miRNAs and TLR expression in bmEos, we identified that two distinct sets of miRNA are likely involved in the expression of TLR4 and TLR13 transcripts (Fig. 7 and Table S7 in File S2). Among these miRNAs, miR-181c shows profound links to TLR4, TLR13 and IL-5Rα; miR-7b also shows the extensive association with major eosinophil factors including TLR4, PU.1, IL-5Rα and CCR3. MiR-669f, with decreased expression, is related to the increased transcripts of both PU.1 and TLR13. These miRNAs with down-regulated profiles are paralleled with their up-regulated targets, suggesting that they may play greater roles in the regulation of eosinophil biology.

Hitherto, there is very limited experimental evidence available for the biological roles of the identified miRNA networks. However, emerging studies show the unique roles of several of miRNAs in controlling cell growth and differentiation and it is likely that these miRNAs, forming networks, may influence the process of eosinophilopoiesis *via* the post-transcriptional regulation. For example, miRNA cluster including let-7e and miR-125a is implicated in hematopoiesis [66]; miR-7b inhibits proliferation of mouse pancreatic β cells [73]; miR-144 together with miR-451 controls erythropoiesis [39]; miR-130a plays key roles in regulating angiogenesis, tumor development and inflammatory disease and also anti-viral responses [74]–[77]. Furthermore, miR-196a has been shown to regulate stem cell proliferation, fibroblast function, angiogenesis and tumor cell growth [78]–[81].

In conclusion, we have utilized a model of bmEos differentiation, which has enabled examination of the expression patterns of miRNAs during eosinophil development. We have analysed these patterns, and have identified miRNAs that may play pivotal roles in driving bmEos maturation and regulating eosinophil anti-infection function. These selected miRNAs can be grouped as networks in order to guide bone marrow cells toward bmEos development in cooperation with cytokine signaling. Furthermore, there are also key intracellular signaling pathways that may be regulated by these miRNAs. To understand how these selected miRNAs interact with their targets, further functional studies with miRNA targeting are required and we will proceed with knock-in and knock-down models. Manipulating the expression of these miRNAs may promote understanding of the underlying mechanisms that are critical for eosinophil biology and ultimately provide therapeutic benefits for treating eosinophil-associated diseases.

## Supporting Information

Figure S1 **Expression of TLR1, TLR2, TLR3, TLR5, TLR7, TLR8, TLR9, TLR11 and TLR12 correlated with the expression of miRNAs that potentially target these transcripts.** Bone marrow cells were cultured as described in the Methods and RNA samples were extracted from day 4 to day14 from cells grown in the presence of IL-5. Expression levels of the above TLRs were determined by qPCR. Data represent three independent eosinophil cell cultures. Values are presented as mean ±SEM (n = 4∼6), *P<0.01 (vs. d4).
(TIF)

Figure S2 **Luciferase activity in lysates of HEK293 cells transfected with constructs encoding the 3′UTR region of GATA-1 and miRNA mimics (miR-378, let-73, miR-200a and miR-429) or scrambled control mimic at the concentrations indicated.** Ctrl = control. n = 6, values represented as mean±SEM. At respective concentration, *P<0.05, miR-378 mimic v.s. Ctrl mimic; **P<0.05, let-7e mimic v.s. Ctrl mimic treatment; # P<0.05, let-7e mimic v.s. Ctrl mimic; ★ P<0.05, let-7e mimic treatment v.s. Ctrl mimic.
(TIF)

Table S1 **Primer sequence for determining mRNA levels by quantitative PCR.**
(DOC)

File S1 **This file includes:** ***Table S2*** **. Annotation of miRNAs with greater than 5 fold changes, as shown in** **Figure 2** **;** ***Table S4*** **.** **Eosinophil related canonical pathways potentially regulated by the miRNAs;** ***Table S5*** **. Eosinophil related canonical pathways potentially regulated by the miRNAs; and** ***Table S9*** **. Pearson correlation test of the miRNAs and their targets.**
(XLSX)

File S2 **This file includes:** ***Table S3*** **: Potential binding sites between GATA1, PU.1 and their respective miRNAs;** ***Table S6*** **: Potential binding sites between IL-5Rα, CCR3 and their respective miRNAs; and** ***Table S7*** **: Potential binding sites between TLR4, TLR13 and their respective miRNAs.**
(DOCX)

Method S1 **Luciferase reporter assay was described.**
(DOCX)
