# Supplementary figures and images for: Expression Profiling of Differentiating Eosinophils in Bone Marrow Cultures Predicts Functional Links between MicroRNAs and Their Target mRNAs
Source: PLoS One. 2014 May 13;9(5):e97537. doi: 10.1371/journal.pone.0097537 (PMC4019607; doi:10.1371/journal.pone.0097537)

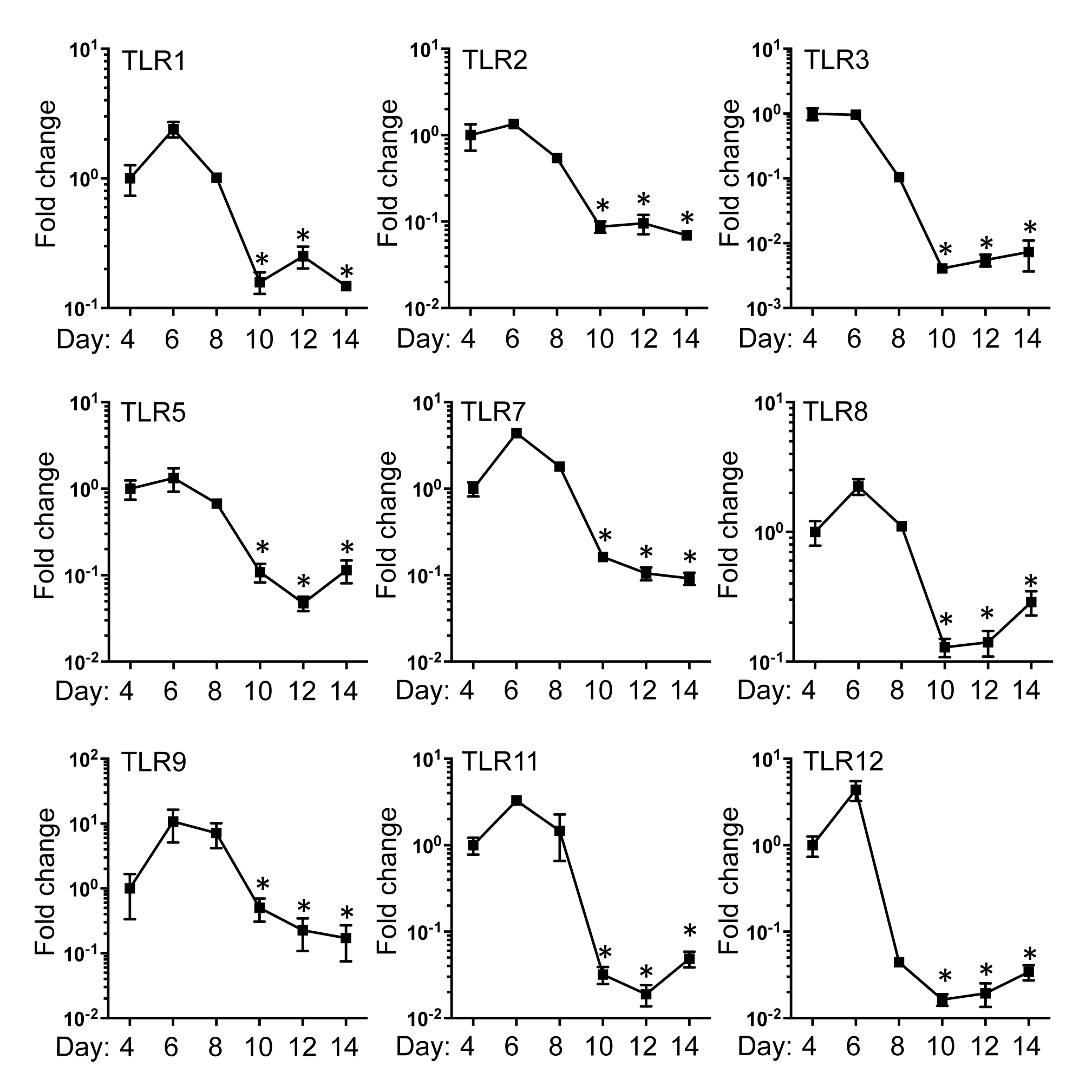

Supplement: Figure S1 — Expression of TLR1, TLR2, TLR3, TLR5, TLR7, TLR8, TLR9, TLR11 and TLR12 correlated with the expression of miRNAs that potentially target these transcripts. Bone marrow cells were cultured as described in the Methods and RNA samples were extracted from day 4 to day14 from cells grown in the presence of IL-5. Expression levels of the above TLRs were determined by qPCR. Data represent three independent eosinophil cell cultures. Values are presented as mean ±SEM (n = 4∼6), *P<0.01 (vs. d4). (TIF) [file pone.0097537.s001.tif]

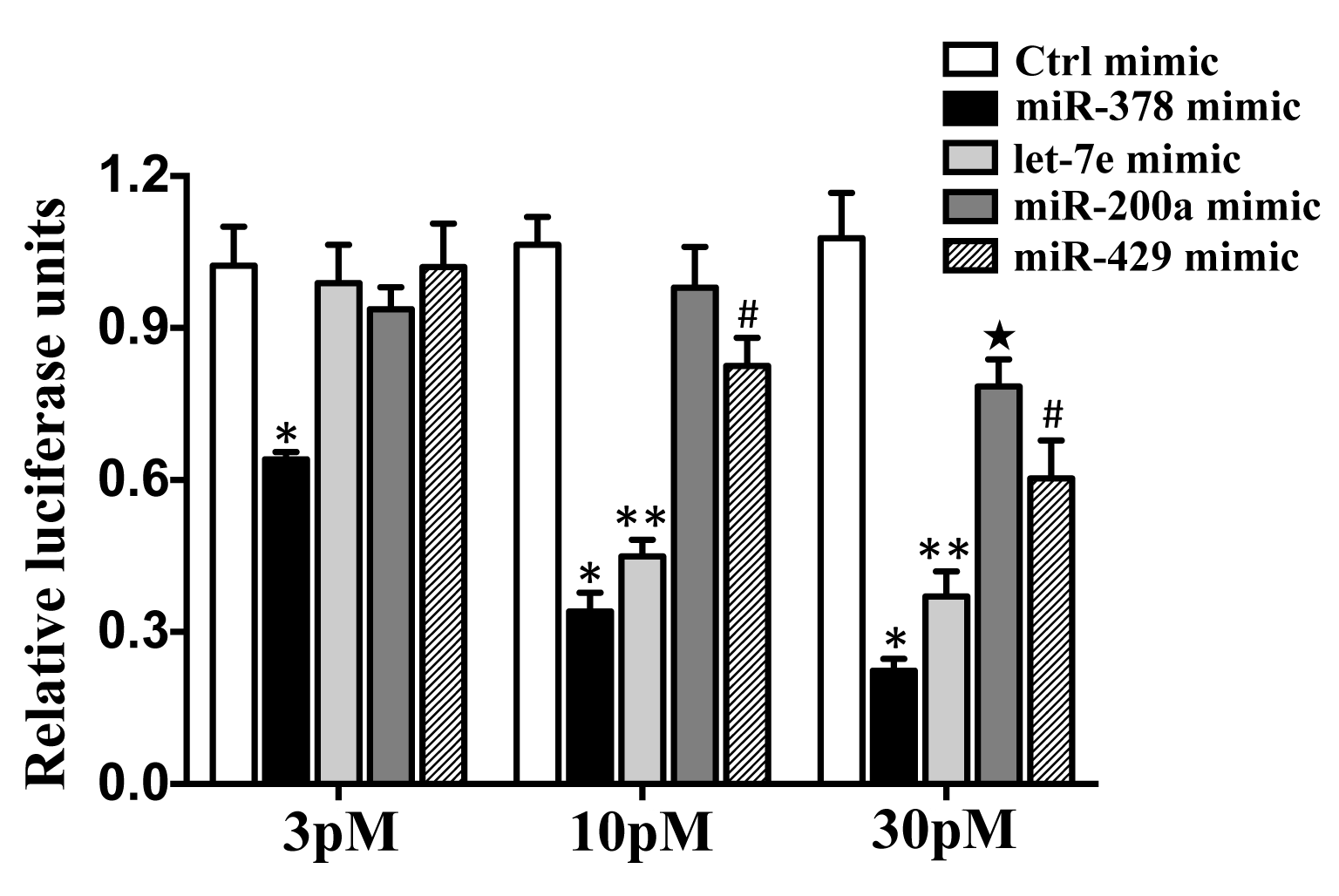

Supplement: Figure S2 — Luciferase activity in lysates of HEK293 cells transfected with constructs encoding the 3′UTR region of GATA-1 and miRNA mimics (miR-378, let-73, miR-200a and miR-429) or scrambled control mimic at the concentrations indicated. Ctrl = control. n = 6, values represented as mean±SEM. At respective concentration, *P<0.05, miR-378 mimic v.s. Ctrl mimic; **P<0.05, let-7e mimic v.s. Ctrl mimic treatment; # P<0.05, let-7e mimic v.s. Ctrl mimic; ★ P<0.05, let-7e mimic treatment v.s. Ctrl mimic. (TIF) [file pone.0097537.s002.tif]
