# Supplementary material for: Expression Profiling of Differentiating Eosinophils in Bone Marrow Cultures Predicts Functional Links between MicroRNAs and Their Target mRNAs
Source: PLoS One. 2014 May 13;9(5):e97537. doi: 10.1371/journal.pone.0097537 (PMC4019607; doi:10.1371/journal.pone.0097537)
Supplement: Table S1 — Primer sequence for determining mRNA levels by quantitative PCR. (DOC) [file pone.0097537.s003.doc]

**Table S1: Primer pairs**

| **Primer Name** | **Direction** | **Sequence (5’-3’)** |
| --- | --- | --- |
| HPRT | Forward | AGGCCAGACTTTGTTGGATTTGAA |
|  | Reverse | CAACTTGCGCTCATCTTAGGCTTT |
| IL-5Rα | Forward | GCTCAAGCAAGCGTGCTGCAATCA |
|  | Reverse | CCACCTTCCTGGCATTCTGCAAGGT |
| CCR3 | Forward | CTACTGGACTCATAAAGGACTTAGCA |
|  | Reverse | TGAGGATCAACACAACCATCATGTT |
| MBP | Forward | GGGTGACTCTGGATGCAAGACCTGT |
|  | Reverse | CTGCCCAGCAGCCCAGTATGCAA |
| GATA1 | Forward | CGCTCCCTGTCACCGGCAGTGCTTA |
|  | Reverse | CCGCCACAGTGGAGTAGCCGTTGCT |
| GATA2 | Forward | CTCCCGACGAGGTGGATGTCTT |
|  | Reverse | CCTGGGCTGTGCAACAAGTGTG |
| c/EBPε | Forward | GACCTACTATGAGTGCGAGCCT |
|  | Reverse | CTGCTACCCTCATCAAGGGTGT |
| Sfpi1 (PU.1) | Forward | TGTCCACAACAACGAGTTTGAGAA |
|  | Reverse | GGGACAAGGTTTGATAAGGGAAGC |
| TLR1 | Forward | TTTGTCCCACAATGAGCTAAAGG |
|  | Reverse | TTCTTTGCATATAGGCAGGGC |
| TLR2 | Forward | CTCTTCAGCAAACGCTGTTCT |
|  | Reverse | GGCGTCTCCCTCTATTGTATTG |
| TLR3 | Forward | TGTCCACAACAACGAGTTTGAGAA |
|  | Reverse | GGGACAAGGTTTGATAAGGGAAGC |
| TLR4 | Forward | GCATCTGCGAGCACGAGACGCT |
|  | Reverse | CGCCTTGGCCTTCTCCTGCTGT |
| TLR5 | Forward | GGAACATATGCCAGACACATCTGT |
|  | Reverse | GCTATCCTGCCGTCTGAAGAACA |
| TLR6 | Forward | AGCCAAGACAGAAAACCCATC |
|  | Reverse | GGGGTCATGCTTCCGACTAT |
| TLR7 | Forward | ATGTGGACACGGAAGAGACAA |
|  | Reverse | ACCATCGAAACCCAAAGACTC |
| TLR8 | Forward | TTCCAGAAGCTATCCTTGTGACG |
|  | Reverse | CATGCAGTTGACGATGGTTGC |
| TLR9 | Forward | TCCTCCATCTCCCAACATGGTTCT |
|  | Reverse | CAGGTGGTGGATACGGTTGGAGAT |
| TLR11 | Forward | TCCCTGATTGCATCATAGCAGA |
|  | Reverse | GGGCCGAGGTACAGAATGG |
| TLR12 | Forward | CCTGGTCTCCCGCTATTTCAC |
|  | Reverse | CCGAGGTACAACTTCCAAGGT |
| TLR13 | Forward | GTTGTAACCTGGATGCCTAAGAC |
|  | Reverse | GGCCTCTGTCAAGTTGGTGA |
