# Supplementary material for: Expression Profiling of Differentiating Eosinophils in Bone Marrow Cultures Predicts Functional Links between MicroRNAs and Their Target mRNAs
Source: PLoS One. 2014 May 13;9(5):e97537. doi: 10.1371/journal.pone.0097537 (PMC4019607; doi:10.1371/journal.pone.0097537)
Supplement: File S2 — This file includes: Table S3 : Potential binding sites between GATA1, PU.1 and their respective miRNAs; Table S6 : Potential binding sites between IL-5Rα, CCR3 and their respective miRNAs; and Table S7 : Potential binding sites between TLR4, TLR13 and their respective miRNAs. (DOCX) [file pone.0097537.s005.docx]

**Table S3: Potential binding sites between GATA1, PU.1 and their respective miRNAs**

| **Target Gene** | **microRNAs** | **Binding Sites** |
| --- | --- | --- |
| GATA1,  NM_008089 | miR-378 | 3' ggaAGAC-UGAGG--U---UCAGGUCa 5'  \|:\|\| \|:\|\|\| \| \|\|\|\|\|\|\|   256:5' ggcUUUGAAUUCCUUAUGUAGUCCAGa 3' |
|  |  | 3' ggAAGACUGAGGUUC-AGGUCa 5'  \| \| \|\|\|\| \|\|\| \|\|\|\|\|   275:5' agUCCAGACU--AAGCUCCAGc 3' |
|  | let-7e | 3' uuGAUAUGUUGGAGGAUGGAGu 5'  :\| \| \| :\|\|\| \|\|\|\|\|\|\|   118:5' caUUCUCCUGCCU-CUACCUCc 3' |
|  | miR-200a | 3' ugUAGCAAUGGUCUGUCACAAu 5'  :: \|\| \| :\| \|\| \|\|   145:5'cugagAUU-CAGGCAUGUAUUg 3' |
|  | miR-429 | 3' ugcCGUAAUGGUCUGUCAUAAu 5'  \| \| \|:\| \|:\|\|\|\|\|\|\|  1361:5' agaGAAGAAUC-GGCAGUAUUu 3' |
| GATA2,  NM_008090 | miR-132 | 3' gcugguaccgacaucUGACAAu 5'  \|\|\|\|\|\|  1492:5' uccuuuaaagugaauACUGUUa 3' |
|  | miR-193b | 3' ucgcccugaaACACCCGGUCAa 5'   \| \| \|\|\|\|\|\|\|   762:5' cagccugugcUCUAGGCCAGUc 3' |
|  |  | 3' ucgcccUGAAACACCC-GGUCAa 5'  ::\|\|\| \|\|\|\| \|\|\|\|\|   915:5' aaacucGUUUUUUGGGUCCAGUc 3' |
|  | miR-144 | 3' ucauguaguagauAUGACAu 5'  \|\|\|\|\|\|   177:5' cuugugccgccauUACUGUg 3' |
|  |  | 3' ucAUGUAGUAGAUAUGACAu 5'  \| :\| :\| \|\|\|\|\|\|\|  1493:5' ccUUUAAAGUGAAUACUGUu 3' |
|  | miR-363 | 3' auguCUACCUA--UGG--CACGUUAa 5'   \| \|\| :\| \|\|\| \|\|\|\|\|\|\|  1270:5' uuguGCUGAGUCAACCAAGUGCAAUa 3' |
|  | miR-200a | 3' ugUAGCAAUGGU--CUGUCACAAu 5'  \| :\|\|\| :\|\| \|\| \|\|\|\|\|\|   247:5' aaAGUGUUUUCACGGAGAGUGUUu 3' |
|  | miR-429 | 3' ugcCGUAAUGGUCUGUCAUAAu 5'  \| \| \|:\| \|:\|\|\|\|\|\|\|  1361:5' agaGAAGAAUC-GGCAGUAUUu 3' |
| PU.1  NM_011355 | miR-669f | 3' uaugcacacacACAUACAUAUAc 5'  \| \| \|\|\|\|\|\|\|   259:5' gcgcuggcaccUUUUUGUAUAUu 3' |
|  | miR-429 | 3' ugccgUAAUGGUCUGUC-AUAAu 5'   \|\|\|\|:: \|\|\|\|\| \|\|\|\|   337:5' guaaaAUUAUU-GACAGCUAUUc 3' |
|  | miR-155 | 3' ugGGGAUAG-UGUUAAUCGUAAUu 5'   \|\|\| : \| :\| \| \|\|\|\|\|\|\|\|   28:5' gaCCCCGCCGGCCA-UAGCAUUAa 3' |
|  | miR-7b | 3' uguuguuuuaguguucAGAAGGu 5'   \|\|\|\|\|\|   284:5' augcuuuuuaaaaagcUCUUCCu 3' |
| c/EBPε  NM_207131 | miR-130a | 3' uacgggaaaauuguaACGUGAc 5'  \|\|\|\|\|\|   144:5' uguggcugaauaaacUGCACUg 3' |
|  | miR-152 | 3' gguucaagacaguACGUGACu 5'  \|\|\|\|\|\|\|   146:5' uggcugaauaaacUGCACUGu 3' |
|  | miR-194 | 3' agguguaccucaacGACAAUGu 5'  \|\|\|\|\|\|\|   149:5' cugaauaaacugcaCUGUUACu 3' |

**Table S6: Potential binding sites between IL-5R**α**, CCR3 and their respective miRNAs**

| **Target Gene** | **microRNAs** | **Binding Sites** |
| --- | --- | --- |
| IL-5Rα,  NM_008370 | miR-362-5p | 3' uaagugUGGAUCCAAGGUUCCUAa 5'  \| \|\| \| \|:\|\|\|\|\|\|\|  1154:5' gacagaAGCUUG--UUCAAGGAUu 3' |
|  |  | 3' uaaguguggauccaaggUUCCUAa 5'   \|\|\|\|\|\|   547:5' cuacagugacaggaggaAAGGAUa 3' |
|  | miR-669b | 3' uguaCGU-GUACG-UG-UGUUUUGa 5'   \|\|\| \|\| \|\| \|\| \|\|\|\|\|\|\|   892:5' aacaGCAGCA-GCAACAACAAAACa 3' |
|  | miR-486 | 3' gagccccGUCGA-GUCA-UGUCCu 5'  \|\|\|\|\| \|\|\|\| \|\|\|\|\|   537:5' uagcacuCAGCUACAGUGACAGGa 3' |
|  |  | 3' gagccccgucgagucAUGUCCu 5'  \|\|\|\|\|\|   15:5' ucugaaaugaacucaUACAGGa 3' |
|  | miR-7b | 3' ugUUGUUUUA---GUGU----UCAGAAGGu 5'   \|:::\|\|:\| \|\|\|\| :\|\|\|\|\|\|\|  1761:5' gcAGUGAAGUAUCCACACUUUGGUCUUCCu 3' |
|  | miR-467e | 3' ugUAUAUGUACG--AGUGUGAAUa 5'  \| \|\|\| \| \|\| \|\|\|\|\|\|\|\|  1446:5' gaACAUAGAAGCAAACACACUUAu 3' |
|  | miR-181c | 3' ugAGUGGCUGUCCAACUUACAa 5'  \|::\|:\|: \| \| \|\|\|\|\|\|  1249:5' gaUUGCUGG-AUCUAGAAUGUa 3' |
|  | miR-1896 | 3' gaggagugggugguaGUCUCUc 5'  \|\|\|\|\|\|  1881:5' cccuaaugaaggagcCAGAGAa 3' |
|  |  | 3' gaggagugggugguaGUCUCUc 5'  \|\|\|\|\|\|   203:5' acagccagagcuacaCAGAGAa 3' |
| CCR3  NM_009914 | miR-467a | 3' gcGUAUAUG--UACGUCCGUGAAu 5'   \| \| \|\|\| \| : \|\|\|\|\|\|\|  1533:5' agCCUUUACUUAACUUGGCACUUu 3' |
|  |  | 3' gcguaUAUGUACGUCCGUGAAu 5'   \|\| \| \|:: \|\|\|\|\|\|  1050:5' ucuggAUUGAAGUGUGCACUUa 3' |
|  | miR-1896 | 3' gaggaguggGUGGUAGUCUCUc 5'   ::\| \|\|\|\|\|\|\|  1188:5' uaacugugaUGCGUUCAGAGAc 3' |
|  |  | 3' gaggagugggugguaGUCUCUc 5'   \|\|\|\|\|\|   1:5' ----------uugggCAGAGAa 3' |
|  | miR-193b | 3' ucgcCCUG-AAACACCCGGUCAa 5'   \|\|\|\| \| \|\| \|\|\|\|\|\|\|   179:5' uagaGGACAUGUGCUGGCCAGUu 3' |
|  |  | 3' ucgcccugaaacaccCGGUCAa 5'  \|\|\|\|\|\|   560:5' cacucuccucauaauGCCAGUu 3' |
|  | miR-292-5p | 3' guUUUCUCGGGGGUCAAACUCa 5'  :\| \|\|\| \| \| \|\|\|\|\|\|\|  1884:5' guGAUGAGACAGCUGUUUGAGg 3' |
|  |  | 3' guuuUCUCGGGGGUCA-AACUCa 5'  :\|:\|\|\| :\| \|\| \|\|\|\|\|  597:5' ggcuGGGGCCAUCUGUAUUGAGa 3' |
|  |  | 3' guuuucucgggggucAAACUCa 5'   \|\|\|\|\|\|  1618:5' uaaccaugacaacuaUUUGAGc 3' |
|  | miR-467b | 3' guaUAUGUACGUCCGUGAAUg 5'  \|\| \| \|:: \|\|\|\|\|\|\|  1052:5' uggAUUGAAGUGUGCACUUAu 3' |
|  |  | 3' guAUAUGUACG----UCCGUGAAUg 5'  \|\|\|\|\|\|\|\|: \| \|\|\|\|\|\|  259:5' guUAUACAUGUUCUGAUUCACUUAa 3' |
|  |  | 3' guauauguacguccGUGAAUg 5'  \|\|\|\|\|\|   5:5' gcagagaaaauuguCACUUAu 3' |
|  | miR-378 | 3' ggaAGAC-UGA-GGUUCAGGUCa 5'   \|\|\|\| \|:\| \|\|\|\|\|\|\|\|   605:5' ccaUCUGUAUUGAGAAGUCCAGg 3' |
|  | miR-467e | 3' uguAUAUGUACGAG----UGUGAAUa 5'  \|\|\|\|\|\|\|\|:\|\| \|\|\|\|\|\|  258:5' aguUAUACAUGUUCUGAUUCACUUAa 3' |
|  |  | 3' uguauauguacgaguGUGAAUa 5'  \|\|\|\|\|\|   4:5' ggcagagaaaauuguCACUUAu 3' |
|  |  | 3' uguaUAUGUACGAGUGUGAAUa 5'   \|\| \| \|: ::\|\|\|\|\|\|  1051:5' cuggAUUGAAGUGUGCACUUAu 3' |
|  | miR-486 | 3' gagccCCGUCGAG--UCAUGUCCu 5'  \|\|:\| \|\|\| \| \|\|\|\|\|\|   694:5' gcacaGGUAACUCCUAUUACAGGa 3' |
|  | miR-421 | 3' cgcgGGUUAAUUACAGACAACUa 5'  \|\|\|:\|\| \| \| \|\|\|\|\|\|   572:5' aaugCCAGUU--U-UAUGUUGAa 3' |
|  | miR-7b | 3' uguuguuuUAGU-GUUCAGAAGGu 5'  :\|\|: :\|\| \|\|\|\|\|\|   629:5' guggugcuGUCGUUAAUUCUUCCu 3' |

**Table S7: Potential binding sites between TLR4, TLR13 and their respective miRNAs**

| **Target Gene** | **microRNAs** | **Binding Sites** |
| --- | --- | --- |
| TLR4,  NM_021297 | miR-130a | 3' uacgGGAAAAUUGUAAC-GUGAc 5'  \|:\|\| \| \|:\|\|\|\| \|\|\|\|   700:5' uauaCUUUGUCAUAUUGUCACUg 3' |
|  | miR-200a | 3' uguaGCAAUG-GUCUGUCACAAu 5'  :\|\| \|\| \|\| \|\|\|\|\|\|\|   753:5' uggaUGUCACAGAGUCAGUGUUa 3' |
|  | miR-539 | 3' ugUGUGGUUCCUAUUAAAG-AGg 5'  \|\|\| \|\| \|\|:\|\|\|\|\| \|\|   836:5' gaACAGAAAUCAUGAUUUCAUCa 3' |
|  | miR-181c | 3' ugaGUGGCUGUCCAACUUACAa 5'  :\|::\| \|\| \|\|\|\|\|\|\|   709:5' ucaUAUUGUCA--CUGAAUGUc 3' |
|  |  | 3' ugagugGCUGU-CCAACUUACAa 5'  :\|\| \| \|\| \|\|\|\|\|\|\|  1025:5' gccuugUGAGAUGGAUGAAUGUu 3' |
|  |  | 3' ugaguggcuguccaACUUACaa 5'  \|\|\|\|\|\|   374:5' ucccuuuuuucuacUGAAUGca 3' |
|  | miR-181d | 3' uggguggcuguuGUUACUUACAa 5'  \|\| \|\|\|\|\|\|\|   706:5' uugucauauuguCACUGAAUGUc 3' |
|  |  | 3' ugggugGCUGUUGUUACUUACAa 5'  :\|\| \| :\|\|\|\|\|\|\|\|  1025:5' gccuugUGAGAUGGAUGAAUGUu 3' |
|  |  | 3' uggguggcuguuGUUACUUACaa 5'   :\| \|\|\|\|\|\|   373:5' uucccuuuuuucUACUGAAUGca 3' |
|  | miR-374 | 3' guGAAUCGUCCAACAUAA-UAUa 5'  :\|\|\|\| \|\| \|\|\|\|\| \|\|\|   324:5' gcUUUAGAAG--AGUAUUGAUAg 3' |
|  | miR-135a | 3' aguGUAUCCUUAUUUUUCGGUAu 5'  \| \| \|\|\|\| \| \|\|\|\|\|\|\|   282:5' ggcCUUUUGAAU-ACAAGCCAUg 3' |
|  | miR-451 | 3' uuGAG-UCAUUACCAUUGCCAAa 5'  :\|\| \| \|:\| \|\|\|\|\|\|\|   344:5' guUUCAACUGAACUGAACGGUUu 3' |
|  | miR-7b | 3' uguuguuuuaguguucAGAAGGu 5'  \|\|\|\|\|\|   422:5' cuucauuccaauuucaUCUUCCa 3' |
|  | miR-363 | 3' augucuaccuauggcACGUUAa 5'  \|\|\|\|\|\|   377:5' cuuuuuucuacugaaUGCAAUa 3' |
|  |  | 3' augucuaccuauggcACGUUAa 5'  \|\|\|\|\|\|   22:5' uaaacccagucuguuUGCAAUu 3' |
| TLR13  NM_205820 | miR-669f | 3' uaugcacacacacaUACAUAUAc 5'  :\|\|\|\|\|\|\|  539:5' cuuuccuuccccaaGUGUAUAUa 3' |
|  | miR-421 | 3' cgCGGG--UUAAUUACA---GAC-AACUa 5'  \|\|\|\| \|\|\| \|\|\|\|\| \|\|\| \|\|\|\|  39:5' uuGCCCAAAAUAAAUGUAAGCUGUUUGAc 3' |
|  |  | 3' cgcggguuaauuacaGACAACUa 5'  \|\|\|\|\|\|\|  668:5' aggacacucuccaucCUGUUGAg 3' |
|  | miR-1896 | 3' gaGGAGUGGGUGGUA-GUCUCUc 5'  :\|\|\| \|:\| \|: \| \|\|\|\|\|\|   75:5' acUCUCUCUCUCUCUCCAGAGAc 3' |
|  |  | 3' gaggagugggugguaGUCUCUc 5'  \|\|\|\|\|\|   599:5' gcugccuggaagaagCAGAGAg 3' |
|  | miR-152 | 3' gguucAAGACAGUACGU-GACu 5'  \|\|: \|\| \|\|\|\|\| \|\|\|   756:5' ugggcUUUAGUGAUGCAGCUGu 3' |
|  | miR-125b-5p | 3' ucCAGGGUUCUUGGAC-UGAACa 5'   \|\|\| :::\|\|: \|\|\| \|\|\|\|\|  317:5' ugGUCAUGGGAGACUGCACUUGu 3' |
|  | miR-181d | 3' uggguGGCUGUUGUUACUUACAa 5'  \|\|\|:\| :\| \|\|\|\|\|\|\|\|   19:5' caaacCCGGCUGC-AUGAAUGUu 3' |
|  | miR-181c | 3' ugaguGGCUGUCCAACUUACAa 5'  \|\|\|:\| \| \|\|\|\|\|\|\|   19:5' caaacCCGGCUGCAUGAAUGUu 3' |
|  | miR-546 | 3' cugaGGCACGGUGGUa 5'   \|:\| \|\|\|\|\|\|\|   649:5' ccagCUGAGCCACCAg 3' |
|  | miR-28 | 3' gaguuaucuGAC-ACUCGA-GGAa 5'  \|\|\| \|\|\|\|\|\| \|\|\|   674:5' cucuccaucCUGUUGAGCUGCCUg 3' |
|  |  | 3' gaguuaucugacacuCGAGGAa 5'   \|\|\|\|\|\|   778:5' cuuugaaacauuucuGCUCCUg 3' |
