# Supplementary material for: Expression Profiling of Differentiating Eosinophils in Bone Marrow Cultures Predicts Functional Links between MicroRNAs and Their Target mRNAs
Source: PLoS One. 2014 May 13;9(5):e97537. doi: 10.1371/journal.pone.0097537 (PMC4019607; doi:10.1371/journal.pone.0097537)
Supplement: Method S1 — Luciferase reporter assay was described. (DOCX) [file pone.0097537.s006.docx]

**Supplementary Method**

**Luciferase reporter assay**

To assess miRNA/target interactions, the 3’-UTR of murine GATA-1 were cloned from total mouse genomic DNA (Promega) into the PsiCheck2 dual-luciferase vector (Promega) using the following primers: forward (5’-3’, GTACACAGAATAGCCTTGACCTTGT) and reverse (5’-3’, CCACTTGACACTGACATTTATTTAACC). Resulting clones were sequenced to verify proper sequence identity.

HEK293 cells (2×10^4^ cells/well seeding in 96-well plate) were co-transfected with each dual-luciferase construct (200 ng/ml), with miRNA mimics (miR-378, let-73, miR-200a and miR-429) or control miR-mimic (Qiagen) using HiPerfect transfection reagent (Qiagen) and lysed 48h later using Glo-Lysis buffer (Promega). Both firefly and renilla luciferase activities were measured using the Dual-Glo Luciferase Assay System (Promega) and quantified on a SpectraMax M5 Multimode Microplate Readers (Molecular Devices). Firefly luciferase was normalized to renilla luciferase activity, presented as relative luciferase units.
